# 804 First-in-Human Xenotransplantation US-FDA-Clinical Trials - Complete Wound Closure of Mixed-Depth Burns via Porcine Skin Xenotransplant

**DOI:** 10.1093/jbcr/irae036.344

**Published:** 2024-04-17

**Authors:** Bounthavy F Homsombath, Paul Holzer, Kaitlyn M Rogers, Joshua C Doloff, Steve Gullans, Robert A Kaiser, Harold Swartz, Curtis L Cetrulo, Jr., Linda Scobie, Rod Monroy, Jeremy Goverman

**Affiliations:** JMS Burn Center at Doctors Hospital Augusta, Augusta, GA; Johns Hopkins University, Grantham, NH; XenoTherapeutics Foundation, Madisonville, LA; Johns Hopkins University, Baltimore, MD; AlexisBio, Natick, MA; Xenotherapeutics, Inc, Rochester, MN; Dartmouth Medical School, Hanover, NH; Massachusetts General Hospital, Boston, MA; Glasgow Caledonian University, Glasgow, Scotland; Xenotherapeutics, Grantham, NH; JMS Burn Center at Doctors Hospital Augusta, Augusta, GA; Johns Hopkins University, Grantham, NH; XenoTherapeutics Foundation, Madisonville, LA; Johns Hopkins University, Baltimore, MD; AlexisBio, Natick, MA; Xenotherapeutics, Inc, Rochester, MN; Dartmouth Medical School, Hanover, NH; Massachusetts General Hospital, Boston, MA; Glasgow Caledonian University, Glasgow, Scotland; Xenotherapeutics, Grantham, NH; JMS Burn Center at Doctors Hospital Augusta, Augusta, GA; Johns Hopkins University, Grantham, NH; XenoTherapeutics Foundation, Madisonville, LA; Johns Hopkins University, Baltimore, MD; AlexisBio, Natick, MA; Xenotherapeutics, Inc, Rochester, MN; Dartmouth Medical School, Hanover, NH; Massachusetts General Hospital, Boston, MA; Glasgow Caledonian University, Glasgow, Scotland; Xenotherapeutics, Grantham, NH; JMS Burn Center at Doctors Hospital Augusta, Augusta, GA; Johns Hopkins University, Grantham, NH; XenoTherapeutics Foundation, Madisonville, LA; Johns Hopkins University, Baltimore, MD; AlexisBio, Natick, MA; Xenotherapeutics, Inc, Rochester, MN; Dartmouth Medical School, Hanover, NH; Massachusetts General Hospital, Boston, MA; Glasgow Caledonian University, Glasgow, Scotland; Xenotherapeutics, Grantham, NH; JMS Burn Center at Doctors Hospital Augusta, Augusta, GA; Johns Hopkins University, Grantham, NH; XenoTherapeutics Foundation, Madisonville, LA; Johns Hopkins University, Baltimore, MD; AlexisBio, Natick, MA; Xenotherapeutics, Inc, Rochester, MN; Dartmouth Medical School, Hanover, NH; Massachusetts General Hospital, Boston, MA; Glasgow Caledonian University, Glasgow, Scotland; Xenotherapeutics, Grantham, NH; JMS Burn Center at Doctors Hospital Augusta, Augusta, GA; Johns Hopkins University, Grantham, NH; XenoTherapeutics Foundation, Madisonville, LA; Johns Hopkins University, Baltimore, MD; AlexisBio, Natick, MA; Xenotherapeutics, Inc, Rochester, MN; Dartmouth Medical School, Hanover, NH; Massachusetts General Hospital, Boston, MA; Glasgow Caledonian University, Glasgow, Scotland; Xenotherapeutics, Grantham, NH; JMS Burn Center at Doctors Hospital Augusta, Augusta, GA; Johns Hopkins University, Grantham, NH; XenoTherapeutics Foundation, Madisonville, LA; Johns Hopkins University, Baltimore, MD; AlexisBio, Natick, MA; Xenotherapeutics, Inc, Rochester, MN; Dartmouth Medical School, Hanover, NH; Massachusetts General Hospital, Boston, MA; Glasgow Caledonian University, Glasgow, Scotland; Xenotherapeutics, Grantham, NH; JMS Burn Center at Doctors Hospital Augusta, Augusta, GA; Johns Hopkins University, Grantham, NH; XenoTherapeutics Foundation, Madisonville, LA; Johns Hopkins University, Baltimore, MD; AlexisBio, Natick, MA; Xenotherapeutics, Inc, Rochester, MN; Dartmouth Medical School, Hanover, NH; Massachusetts General Hospital, Boston, MA; Glasgow Caledonian University, Glasgow, Scotland; Xenotherapeutics, Grantham, NH; JMS Burn Center at Doctors Hospital Augusta, Augusta, GA; Johns Hopkins University, Grantham, NH; XenoTherapeutics Foundation, Madisonville, LA; Johns Hopkins University, Baltimore, MD; AlexisBio, Natick, MA; Xenotherapeutics, Inc, Rochester, MN; Dartmouth Medical School, Hanover, NH; Massachusetts General Hospital, Boston, MA; Glasgow Caledonian University, Glasgow, Scotland; Xenotherapeutics, Grantham, NH; JMS Burn Center at Doctors Hospital Augusta, Augusta, GA; Johns Hopkins University, Grantham, NH; XenoTherapeutics Foundation, Madisonville, LA; Johns Hopkins University, Baltimore, MD; AlexisBio, Natick, MA; Xenotherapeutics, Inc, Rochester, MN; Dartmouth Medical School, Hanover, NH; Massachusetts General Hospital, Boston, MA; Glasgow Caledonian University, Glasgow, Scotland; Xenotherapeutics, Grantham, NH; JMS Burn Center at Doctors Hospital Augusta, Augusta, GA; Johns Hopkins University, Grantham, NH; XenoTherapeutics Foundation, Madisonville, LA; Johns Hopkins University, Baltimore, MD; AlexisBio, Natick, MA; Xenotherapeutics, Inc, Rochester, MN; Dartmouth Medical School, Hanover, NH; Massachusetts General Hospital, Boston, MA; Glasgow Caledonian University, Glasgow, Scotland; Xenotherapeutics, Grantham, NH

## Abstract

**Introduction:**

Currently there is no treatment than can provide complete and durable wound closure of mixed-depth burn injuries, other than intact human skin, which has several drawbacks. An alternative would be a significant advancement in burn care and a meaningful benefit to patient care.

We report here the results of the first-in-human, US-FDA xenotransplantation clinical trial: the use of skin xenotransplants containing live (i.e., non-terminally sterilized, non-xenograft) epidermal and dermal tissues derived from genetically altered, pathogen-free porcine donors to provide complete and durable wound closure of mixed-depth burn wounds.

**Methods:**

22 patients with mixed-depth burn wounds were enrolled in Phase 1/2/2b US-FDA clinical trials to evaluate the safety and efficacy of skin xenotransplants to provide complete and durable wound closure in a side-by-side comparison with human allograft.

**Results:**

To date, there have been zero adverse events including any xeno-related pathologies. In all patients, complete and durable wound closure was demonstrated at all xeno-treatment sites. In 3 cases, mixed-depth burns treated with the skin xenotransplant did not require autografting.

**Conclusions:**

These clinical data demonstrate the ability of a live, genetically altered skin xenotransplant to facilitate complete and durable wound closure in mixed-depth burn wounds, and that the use of skin xenotransplants may potentially reduce the need for autografting in the treatment of mixed-depth burns. These claims will be evaluated in a US-FDA Phase 3 clinical trial.

**Applicability of Research to Practice:**

First-in-Human Xenotransplant; Complete, Durable Wound Closure of Mixed, Depth, Full-Thickness Burn Wounds.

